# Sensor-Based Detection of the Severity of Hyperkeratosis in the Teats of Dairy Cows

**DOI:** 10.3390/s18113925

**Published:** 2018-11-14

**Authors:** Susanne Demba, Gundula Hoffmann, Christian Ammon, Sandra Rose-Meierhöfer

**Affiliations:** 1 Department of Engineering for Livestock Management, Leibniz Institute for Agricultural Engineering and Bioeconomy e.V. (ATB), Max-Eyth-Allee 100, 14469 Potsdam, Germany; susanne.demba@gmx.de (S.D.); cammon@atb-potsdam.de (C.A.); 2 Hochschule Neubrandenburg, Department of Agricultural Machinery, University of Applied Science, Brodaer Straße 2, 17033 Neubrandenburg, Germany; rose@hs-nb.de

**Keywords:** sensor, dielectric constant, water content, dairy cow, teat end hyperkeratosis

## Abstract

The aim of this study was to evaluate whether the severity of hyperkeratosis (HK) in the teats of dairy cows can be assessed by a dielectric measurement. The study focused on surveying the occurrence of hyperkeratosis in a total of 241 teats of lactating dairy cows. A scoring system consisting of four categories was used to macroscopically assess the severity of HK. Additionally, the dielectric constant (DC) of all teats with milkability was measured in a double iteration with the MoistureMeterD (Delfin Technologies, Kuopio, Finland) on four different days. The Spearman rank correlation coefficient revealed a negative correlation between the DC and HK score (rs = −0.55 to −0.36). The results of the regression analysis showed that the DC values differed significantly between healthy teat ends (≤2) and teat ends with HK (≥3). Thus, the non-invasive measurement of DC provides a promising method of objectively assessing the occurrence and severity of HK.

## 1. Introduction

Teat end hyperkeratosis (HK) is defined as a thickened smooth keratin ring or as extending fronds of keratin around the teat canal orifice [1]. The occurrence of HK greatly impacts milk production. The severity of HK is an issue of importance because teat condition is connected with the capability to defend against mastitis pathogens [2]. Furthermore, serious HK as well as a relatively higher roughness of the teat end both increases the risk of udder diseases [3,4,5]. Milk from non disinfected teats with an HK score higher than one have a larger content of somatic cells [6]. The prevalence of HK has been associated with many factors, such as season [7,8] and teat morphology [7,9] as well as milking traits such as milk flow [7], parity [7,9], and days in milk [8]. Milking technique may also play a role in HK formation [10,11,12].

Although HK is of substantial economic and animal health concern, there is currently no appropriate sensor-based method to objectively quantify the severity of HK. Until now the scoring systems of Mein et al. [13] and those of Neijenhuis, Mein, Reinemann, Hillerton, Farnsworth, Baines, Hemling, Ohnstad, Cook, Morgan, and Timms [4] were mostly used to evaluate the influence of biological or technical factors on HK.

Therefore, this study was conducted to assess the use of the dielectric constant (DC) of the teat ends as a possible sensor-based method for monitoring HK. Measuring the DC of biological tissue is normally applied in human medicine. With the help of DC measurements, local changes in water content in the skin and subcutaneous fat could be measured for the early diagnosis of diseases that involve changes in tissue water content. The measurement of the DC provides an instantaneous and non-invasive measurement [14,15].

Measuring the DC is often used to investigate lymphedema [16,17,18], cutaneous edema, tissue fluid status [19,20,21], radiotherapy [22], burns, thermal injury [23], wound healing [24,25], and weight loss [26]. The measurement of DC has been used in the field of agriculture as well. For instance, Hoffmann, et al. [27] used the measurement of DC to monitor the prevalence and severity of foot pad dermatitis in broiler chickens.

The aim of the present study was to validate the potential of measuring the DC of teat ends to evaluate the severity of HK in dairy cows. Therefore, the relationship between the measured values of the DC and the corresponding HK scores of the teats was investigated.

## 2. Materials and Methods

### 2.1. Animals and Study Design

The study was conducted on a commercial dairy farm in Brandenburg, Germany. On four non-consecutive measurement days within six weeks, 241 randomly selected teats of lactating cows were first assessed by visual observations to determine the HK score of each teat. Afterward, the DC of the teat orifice was measured. In general, two teats per cow were examined, if possible one front and one rear teat. Only one teat was examined in one cow, because this cow did not stand still during the measurements and reacted aggressively to the measuring person. Three and four teats were assessed in two cows, respectively, because the teats of these cows were evaluated with an HK score of 4 and not many teats were scored with 4 overall. Thus, the DC of all teats with an HK score of 4 was measured to get a more even distribution of the HK scores. So the decision to investigate more or less than two teats per cow depends on the behavior of the cow during the measuring procedure and the given HK score. None of the examined teats were measured repeatedly. The scoring and the DC measurement were done by one person each to maintain objectivity. The teats were scored once because it was assumed that no new HK would develop during the experimental period. The lactating cows were between their first and sixth parity and ranged from 7 to 438 days in milk. At the time of investigation, the herd had an average milk yield of 30.4 ± 8.38 kg per cow per day, with a mean fat content of 3.95 ± 0.76% and an average protein content of 3.60 ± 0.45%. The mean somatic cell count of the investigated cows was 220,000 cells/mL. The cows were milked twice a day in two separate 2 × 8 herringbone milking parlors equipped with conventional milking clusters (HarmonyTM, DeLaval, Glinde, Germany). For both milking parlors, the machine vacuum was adjusted to 42 kPa, and the cluster weight was 2100 g, with a claw volume of 360 mL. Alternate pulsation was used with a pulsation rate of 60 min^−1^ and a pulsation ratio of 65:35. Teat cups were detached automatically when the milk flow at the udder level fell below 300 g min^−1^. The teat cups were equipped with monoblock liners made of rubber, which had a shaft diameter of 23 mm.

### 2.2. Data Collection

#### 2.2.1. Classification of Hyperkeratosis

Teat end condition was evaluated during the evening milking period using criteria established by Mein, Neijenhuis, Morgan, Reinemann, Hillerton, Baines, Ohnstad, Rasmussen, Timms, Britt, Farnsworth, Cook, and Hemling [13]. The level of hyperkeratosis was classified with scores ranging from one to four as follows: score one indicated a healthy teat without a ring at the teat end; score two indicated a slightly white ring at the teat end; score three indicated a raised and roughened keratin ring, extending up to 3 mm with visible old keratin; and a rating of score four implied a severely roughened keratin ring, extending more than 4 mm with clearly observable old keratin. All the observations were conducted by the same person and were carried out before pre-milking teat preparation. The teats were evaluated before the DC measurements were performed as well. Thus, the scoring person was not influenced by the measured DC values.

#### 2.2.2. Dielectric Measurement

The MoistureMeterD (Delfin Technologies, Kuopio, Finland) with a probe (model XS5) of 10 mm in diameter and an effective measurement depth of 0.5 mm was used to measure the DC of the teat orifice. This device consisted of an electronic control unit and a sensor probe to measure the DC of the skin. The DC is a physical quantity without any unit.

**Figure 1:**
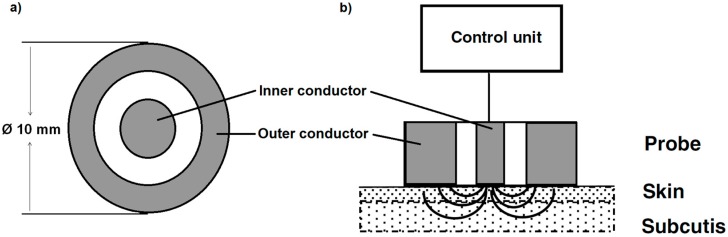
Magnified view of the measuring probe (**a**) and the measuring principle of the MoistureMeterD according to Nuutinen, Ikäheimo and Lahtinen [14] (**b**).

The control unit generates a high-frequency electromagnetic wave of 300 MHz, which is transmitted via an open-ended coaxial probe into the skin and subcutaneous tissues. Here, a majority of the electromagnetic wave energy is absorbed by water, while the rest is reflected (Figure 1). The DC is calculated from the information of the reflected wave, which is directly proportional to the tissue water content [14]. Therefore, the relative changes in the measured DC are also the relative changes in the water content of the measured tissues. As the DC of air is 1 and that of water is 78.5, the values measured on human skin should fall between these values [15]. From the reflected wave, the conductivity can also be calculated, but in the present device, only the DC was determined because it is directly proportional to the tissue water content of the measured skin [14,28,29]. 

**Figure 2:**
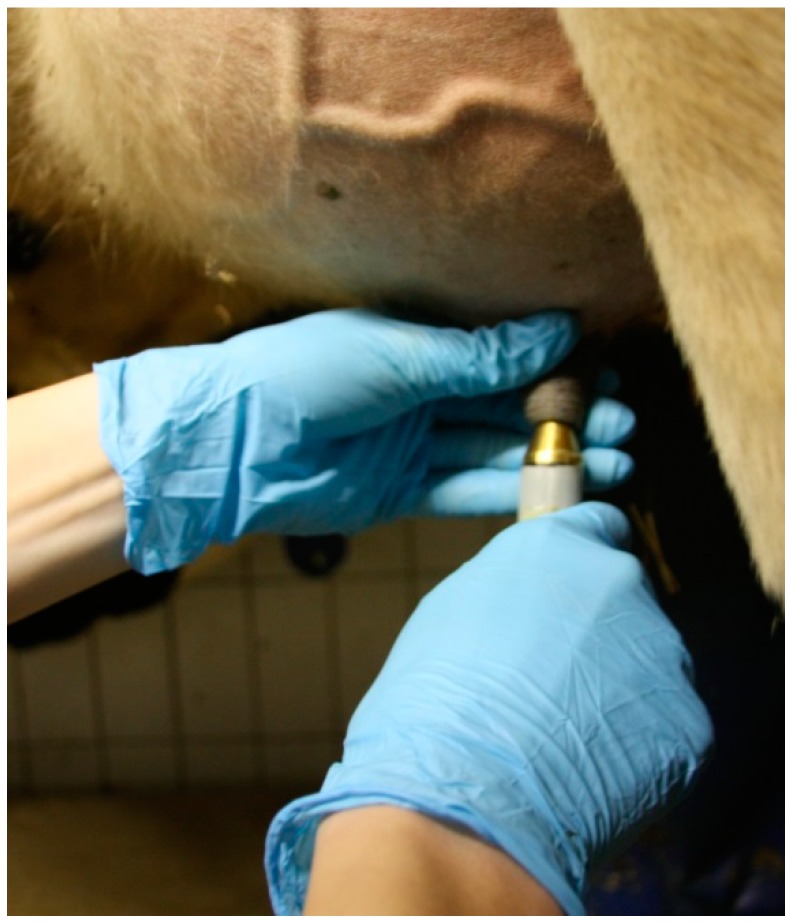
Measuring the dielectric constant at the teat orifice.

The circular probe was manually placed in contact with the skin of the teat orifice (Figure 2). Measuring was automatically started upon skin contact and lasted on average two seconds. Contact between the sensor probe and the skin was regulated by the person who was conducting the measurements, who produced so little pressure that the animals showed no painful reactions. The measurement was repeated two times per teat, except in cases of failed measurements, for which a third measurement was performed. Afterward, the probe was cleaned with a dry cloth. Before pre-milking teat preparation, the same person took all the measurements with the MoistureMeterD to ensure standardized conditions.

In five cows, ten measurements were taken shortly after each other on one teat to test the repeatability of the DC values. Then the coefficient of variation of the measured values per cow was calculated. The measured DC values were considered repeatable if the coefficient of variation was less than 10%.

### 2.3. Statistical Analysis

The Spearman rank coefficient (rs) was used to estimate the correlation between the DC value and the HK score. For the statistical analysis, the possible inference of the DC on the HK score was tested with a generalized linear model approach that assumed a binomial distribution with a logit link function. For this purpose, the median DC of the measurements on each teat was used. A multinomial distribution model with a cumulative logit link function [30] could not be used because the assumption of proportional odds between scores was violated: Therefore, a sequential logit model was used. Given the observed score y_ijkms_ for the ijkm-th teat with scores ranging from 1 to *s*, with s = 4, binary responses were created as follows: (a) score = 1 versus score ≥ 2; (b) score ≤ 2 versus score ≥ 3; (c) score ≤ 3 versus score = 4. Models (a) through (c) examined the ratio between the probability of observing score r or lower and the probability of observing scores higher than r with logit Lr for a maximum r of *s* − 1:

(1) $$L_{r}=\ln\left(\frac{P\left(Y\leq r\right)}{P\left(Y>r\right)}\right)=\ln\left(\frac{P\left(Y\leq r\right)}{1-P\left(Y\leq r\right)}\right)=\theta_{r}+\eta_{r}$$

where θ_r_ is the threshold value for score r and η_r_ is a linear predictor. The linear predictor η_r_ is calculated as follows:

η_ij_ = μ + ax + b_i_ + ε_ij_ (2)

where μ is the general mean; a is the regression coefficient for DC value x; b_i_ is the random effect for cow i; ε_ij_ is the independent logistically distributed residual.

The model was fitted using the GLIMMIX procedure of the SAS 9.4 software package (SAS Institute Inc., Cary, NC, USA). The null hypothesis was that the DC would have no significant influence on the HK score, and all tests were carried out at a significance level of α = 0.05.

## 3. Results

At the time of this investigation, 52, 110, 66, and 13 of the examined teats had an HK score of 1, 2, 3, and 4, respectively. The measured DC values ranged from 10.5 to 52.4. Table 1 shows the sample size, the mean, the standard deviation, the minimum, the 25%-quantile, the median, the 75%-quantile, and the maximum of the measured DC values for each HK score. 

The results of the descriptive statistics lead to the assumption that the mean DC values as well as the values within the 25%-, 50%-, and 75%-quantiles decreased with higher HK scores. This implication is confirmed by the calculation of the Spearman rank correlation coefficient.

**Figure 3:**
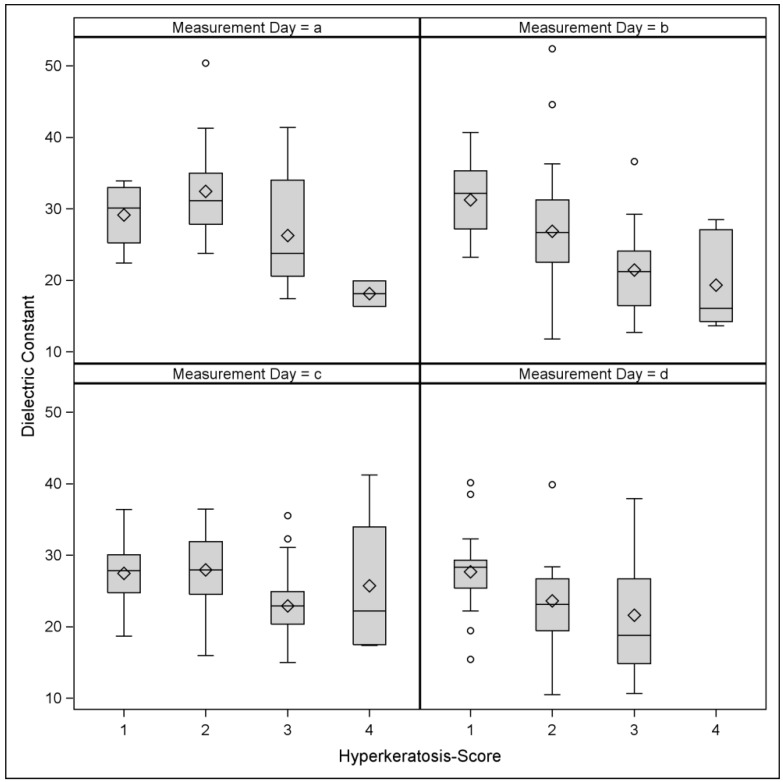
Distribution of the dielectric constant values across the four hyperkeratosis scores on each measurement day.

The values of DC decreased monotonically with increasing HK score (Figure 3). The Spearman rank correlation between the two traits was rs = −0.43 (*p* = 0.005), rs = −0.55 (*p* < 0.001), rs = −0.36 (*p* = 0.001) and rs = −0.39 (*p* = 0.02) on measurement day A, B, C, and D, respectively. Therefore, during all the measurement days, high HK scores were more likely to occur when the DC values were relatively lower, while low HK scores were more frequent when the DC values were high.

The F-tests showed a significant influence of the DC value (*p* < 0.0001) on the HK score when the HK score ≤ 2 (Table 2). No significant influence of the DC value on the HK score was found when the HK score = 1 and when the HK score ≤ 3. The measured DC values showed overlaps among the investigated HK scores.

**Figure 4:**
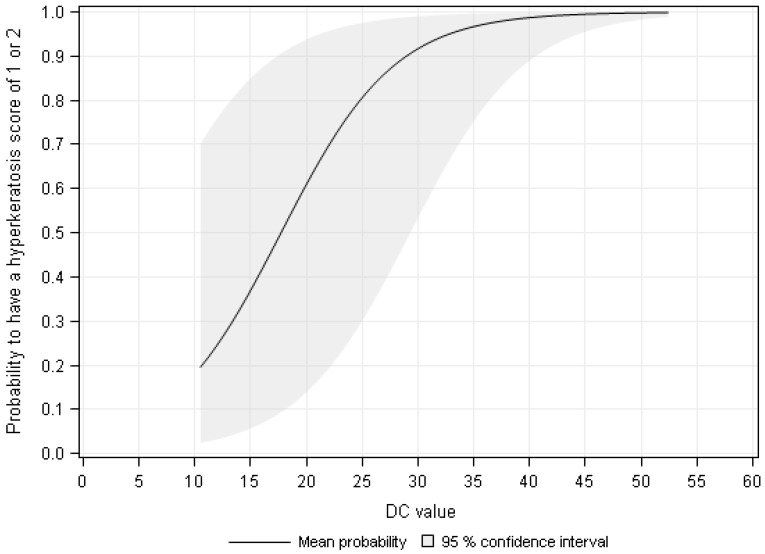
The probability to have a hyperkeratosis score of 1 or 2 depending on the measurement value of the dielectric constant (DC).

The results of the F-test showed an increasing probability to have an HK score of 1 or 2 with increasing DC value (Figure 4). The probability to have an HK score of 1 or 2 was 96.5%, 98.5%, and 100% at a measured DC value of 35, 45, and 50, respectively.

The measured DC values were considered repeatable because the coefficient of variation was, in any case, less than 10% (2.1–9.5%).

## 4. Discussion

The evaluation of teat end HK scores with an external scoring system based on visual observation, according to Mein, Neijenhuis, Morgan, Reinemann, Hillerton, Baines, Ohnstad, Rasmussen, Timms, Britt, Farnsworth, Cook, and Hemling [13] or to Neijenhuis, Mein, Reinemann, Hillerton, Farnsworth, Baines, Hemling, Ohnstad, Cook, Morgan, and Timms [4], is commonly used to assess the severity of HK and to evaluate milking technique. Based on this observational technique, people who score teat ends should be trained, or only one person should score per study. In contrast, the sensor technique used in the present investigation allowed for an objective assessment of the severity of teat end HK. The advantages of the sensor-based method are the objectivity of the measurement results, the independence of the method from light conditions, and the fact that each person could evaluate teat end HK using this method. However, currently, this method is limited by the fact that an assistant needs to hold the control unit, and it is not clear how the relative humidity in the parlor influences the measurement results. Furthermore, a pressure sensor within the probe would also be useful to standardize the pressure used for ensuring the contact between the probe and the teat orifice. Standardized sensor probe pressure during the measurements would ensure comparable results among different studies and among different people who perform the measurements. However, only a little pressure was necessary to conduct the measurements.

The HK scoring system used in the present investigation consisted of four categories based on the presence of a smooth or rough ring around the teat orifice [13]. This scoring system has proven to be useful for analyzing the connection between HK scores and DC values. However, overlaps of the DC values occurred among the four HK scores used in the present investigation. In further studies, more animals should be examined over a longer period of time and repeated measurements should be made on the same teats. Furthermore, histopathological examinations of the HK should be taken into account because the evaluation of scores based only on visual observations are poor indicators of histopathological lesions [31]. A histological investigation of the HK would also help to clarify the interaction of the HK with the electromagnetic wave of the MoistureMeterD because, according to Lahtinen, et al. [32], different skin layers differ in their dielectric properties.

In the present investigation, the measured DC values were on average 26.4. The values for human skin were approximately 36 [14]. The anatomical and physiological differences between human and bovine teat skin (e.g., thickness, tissue water content) could explain the different DC values. Another reason for this result could be the measurement depths. While the sensor probe used in the present investigation had an effective measurement depth of 0.5 mm, Nuutinen, Ikäheimo, and Lahtinen [14] used a probe with an effective measurement depth of 2.5 mm. However, the diameter of this probe (23 mm) was too large to be used for measuring the DC on the teat orifice.

The comparison of DC values and HK scores of the investigated teat orifices indicated that the DC was higher in teat ends with lower scores and vice versa. This finding agrees with the results of Hoffmann, Ammon, Volkamer, Sürie, and Radko [27], who found a negative correlation between DC values and the severity of foot pad dermatitis (FPD) in broiler chickens; specifically the DC values were higher in foot pads with lower FPD scores and were lower in foot pads with higher FPD scores. Hashmi, et al. [33] found lower levels of hydration in hyperkeratotic than in normal foot skin. Alanen, et al. [34] reported that there was always some air between the probe and the skin due to the roughness of the skin. This result might be a reason for the association of lower DCs with severe HK scores, in which crusts covered the teat orifices of the cows. However, the DCs of the four HK scores demonstrated overlap. The results of the regression analysis revealed that DC values differed significantly between healthy teat ends (≤2) and teat ends with HK (≥3). This could be explained by the fact that the visual observation, which is the most commonly used method to assess HK, was used as a gold standard to find correlations between the HK scores and the DC values. The problem with this method is that it is very subjective and sometimes subtleties determine the assignment of the HK score. In an attempt to reduce the disadvantages of this method, the visual observation was always done by the same person. The different distribution of the HK scores in the examined herd may possibly explain this result. 

The DC values measured in the present investigation showed a high variability. There could be several reasons for this variability. First, the manipulation of the teat during the measurements could have resulted in a stimulation of the teat and milk flow could have started in some cows. The fact that not all teats may have been evenly dry as well as the different water content of the teat tissue of individual cows could result in a high variability of the DC values as well. Although the same pressure was attempted to be applied in each measurement, a slight change in this pressure could have changed the measuring depth of the probe and thus led to the high variation in the measured values.

In further studies, the MoistureMeterD compact will be used to validate the DC measurement to objectively assess the occurrence and severity of HK. This device includes a contact pressure sensor and with the help of this sensor a constant pressure could be applied to the teat orifice. Thus, the measuring procedure could be improved. Besides the formation of teat end HK their roughness will also be taken into account in further studies.

## 5. Conclusions

It can be concluded that measuring the DC of the teat orifice is a promising and objective method for assessing the occurrence and severity of HK in dairy cows, but it needs further improvements. This method is particularly suitable to distinguish between healthy teat ends (≤2) and teat ends with HK (≥3). Measuring the DC may be a helpful tool for detecting early teat end irritations; ultimately, this tool may help to improve the teat end condition of dairy cows. However, further studies using more animals and repeated measurements are needed to validate this method and to define exact DC value ranges for the different HK scores. Since this investigation began, Delfin Technologies has further improved the MoistureMeterD. A smaller, handier device that contains a pressure sensor in the probe is now available. This device will be used to validate the DC measurement to objectively assess the occurrence and severity of HK. Once the method has been improved, it could be used as an advisory tool for objective monitoring and early detection of HK by both scientific researchers and animal consulting services. An objective assessment of HK can provide a more detailed analysis of the factors influencing the formation of HK as well.

## Figures and Tables

**Table 1:** The sample size (N), the mean values (Mean), the standard deviation (STD), the minimum (Min), the 25%-quantile (Q1), the median (Median), the 75%-quantile (Q3), and the maximum (Max) of the measured dielectric constant values per hyperkeratosis-score (HK Score).

HK Score	N	Mean	STD	Min	Q1	Median	Q3	Max
1	52	28.98	5.47	15.40	25.60	28.80	32.90	40.70
2	110	27.91	6.95	10.50	23.75	27.85	31.90	52.43
3	66	23.09	6.43	10.65	19.20	22.30	26.15	41.40
4	13	21.00	7.75	13.65	16.10	17.65	25.43	41.25

**Table 2:** Results of the three models (a: score = 1 versus score ≥ 2; b: score ≤ 2 versus score ≥ 3; c: score ≤ 3 versus score = 4) within the generalized linear model approach for the tested effects including the estimate, the standard error, the degree of freedom (DF), the t-value, and the *p*-value.

Model	Effect	Estimate	Standard Error	DF	t-Value	*p*-Value
a	Intercept	7.64	1.97	97	3.88	0.0002
	DC ^1^ value	0.01	0.07	138	0.17	0.8638
b	Intercept	−3.47	1.16	97	−3.00	0.0035
	DC ^1^ value	0.19	0.05	138	4.16	<0.0001
c	Intercept	5.74	2.92	97	1.97	0.0520
	DC ^1^ value	0.17	0.11	138	1.53	0.1277

^1^ Dielectric Constant.
